# Recent research advances in RNA m5C methylation modification in liver diseases

**DOI:** 10.3389/fmolb.2025.1657502

**Published:** 2025-10-17

**Authors:** Wenjuan Chen, Lifan Zhang, Xinyu Gu, Yafeng Liu, Shujun Zhang, Xinjun Hu, Penghui Li

**Affiliations:** ^1^ Department of Infectious Diseases, The First Affiliated Hospital, College of Clinical Medicine, Henan University of Science and Technology, Luoyang, Henan, China; ^2^ Henan Medical Key Laboratory of Gastrointestinal Microecology and Hepatology, Luoyang, China; ^3^ Henan Key Laboratory of Cancer Epigenetics, Cancer Institute, The First Affiliated Hospital, College of Clinical Medicine, Medical College of Henan University of Science and Technology, Luoyang, China; ^4^ Department of Gastrointestinal surgery, The First Affiliated Hospital, College of Clinical Medicine, Henan University of Science and Technology, Luoyang, Henan, China

**Keywords:** liver disease, m5C modification, hepatic pathophysiology, lipid metabolism, HBV, HCV, liver cancer, M5C regulatory factor

## Abstract

RNA m5C methylation refers to the process wherein the 5th carbon atom of cytosine in RNA molecules is methylated by the action of methyltransferase, thus forming 5-methylcytosine (m5C). This crucial epigenetic modification significantly impacts gene expression and various biological processes. The abnormal regulation of this process is closely linked to the occurrence and development of various diseases. The liver is the largest digestive metabolic organ, where numerous critical physiological processes take place. Recent studies have emphasized the unique role of m5C modifications in liver physiology and pathology. This review summarizes the common m5C regulatory factors and their functions, with a particular emphasis on the biological roles of m5C RNA methylation regulators in liver injury, liver immunology, viral hepatitis, fatty liver disease, and liver cancer. Furthermore, it compiles findings on m5C regulators and their inhibitors in the treatment and prognosis of liver diseases.

## 1 Introduction

RNA epigenetic modifications constitute an important mechanism for regulating gene expression. Among them, m5C methylation is an increasingly known RNA modification mechanism that is widely present in various RNA molecules. Its functions include regulating RNA stability, translation, and multiple cellular functions such as proliferation and differentiation (Zhao et al., 2017; Xue et al., 2022). In recent years, owing to the advancement of RNA m5C locus detection methods, m5C has attracted increasing research interest. The m5C methyltransferase family continues to expand, highlighting the crucial role of m5C methylation modification in various diseases. In particular, recent studies have shown that m5C methylation modification has a significant impact on the progression of liver diseases (Chen L. et al., 2019; Mann, 2014). Because the abnormal expression of m5C methyltransferase has a complex relationship with the occurrence, development and treatment response of a variety of liver diseases, they have become the focus of research as prognostic disease markers and potential therapeutic targets (Li O. et al., 2025; Li ZL. et al., 2024; Shi et al., 2025; Huang L. et al., 2024).

The liver is the central metabolic and immune organ of the human body (Heymann and Tacke, 2016; Trefts et al., 2017), where many critical physiological processes, including immune response, lipid regulation, tissue damage and remodeling, are carried out. Therefore, the health of the liver is essential for survival (Russell and Monga, 2018). Liver diseases are responsible for 4% of all deaths worldwide (i.e., 1 in 25 deaths) (Devarbhavi et al., 2023). Among them, Acute-on-Chronic Liver Failure (ACLF) is a significant and unique syndrome that has a high economic burden due to the complex treatment involved (Allen and Kim, 2016; Moreau et al., 2021). In the latest statistics of viral hepatitis-related diseases in 2020, hepatitis B and C viruses led to 1.1 million deaths, far exceeding the number of deaths caused by infectious diseases such as AIDS and malaria. Interestingly, the global resources used to control and eliminate viral hepatitis are far less than those used for these two infectious diseases. Liver cancer is also a major cause of cancer-related deaths. Despite these facts, we are still at a critical period in the understanding and management of liver disease (Devarbhavi et al., 2023).

Studies have found that the m5C methylation affects liver diseases, with an intricate pathological mechanism involved. Therefore, this review discusses the role of m5C modification-related enzymes and other key aspects of liver inflammation, immunity, steatosis, tumorigenesis, and other biological functions along with their clinical significance. Moreover, it summarizes the potential role of m5C regulatory factors and m5C regulatory factor inhibitors in the treatment of liver diseases. Thus, this paper aims to provide a new theoretical framework for the occurrence, development and treatment of liver diseases.

## 2 Molecular regulatory mechanisms of m5C RNA methylation

### 2.1 M5C RNA methylation

Chemical modification stands for the covalent modification of specific chemical groups on biological macromolecules (Proteins, DNA, RNA, sugars, lipids) through chemical reactions to regulate their structure, function or interaction. Over the past 50 years, the greatest number of modifications have been found on proteins and RNA in animal cells (Barbieri and Kouzarides, 2020). On RNA, important effects on gene expression are mainly exerted through chemical changes in RNA bases and ribose. Post-transcriptional RNA modifications, while they do not alter the sequence of the genome, can change gene expression and the function of RNA in various ways, playing an important role in epigenetics (Gu et al., 2023). RNA methylation modification has been proven to be involved in various metabolic-related diseases such as type 2 diabetes and hyperlipidemia. In recent years, m5C RNA methylation has also gradually attracted the attention of researchers and been the subject of study in relation to lipid metabolism and glucose metabolism in the liver (Li Q. et al., 2024). RNA methylation modification plays an important role in epigenetic modification. 170 kinds of modifications have been discovered on RNA, and among these RNA modifications, various methylations account for about 70% of the total (Barbieri and Kouzarides, 2020; Jo et al., 2017; Roundtree et al., 2017; Cui L. et al., 2022).

M5C methylation was discovered on the fifth carbon atom of cytosine in RNA molecules in 1958, and is currently known as a common form of chemical modification, widely distributed in various coding and non-coding RNA (Gu et al., 2023; García-Vílchez et al., 2019; Shi et al., 2023). More than 90,000 m5C sites have been detected in the human genome so far, and these are mainly enriched in the 3′-untranslated region (3'-UTR) or near the translation start codon (Boo and Kim, 2020). The classic method of detecting m5C sites is bisulfite sequencing, while subsequent studies have suggested that there may be a large number of false positive results (Bartee et al., 2021). Hence, scholars have increasingly developed new detection methods (Table 1), such as applying high temperature before bisulfite sequencing or destroying the secondary structure of RNA by formamide treatment to improve the conversion rate from C-T/U and reduce the rate of false positives (Khoddami et al., 2019; Huang et al., 2019). These technological developments have greatly advanced the understanding of the critical role of m5C RNA methylation in the regulation of gene expression, disease progression, and cellular function.

**TABLE 1:** Approaches for the mapping of m5C in RNA.

Detection method	Experimental principle	Advantages	Disadvantages	References
BS-Seq	Converts unmethylated cytosine (C) to uracil (U) via bisulfite	Single-base resolution High sensitivity Non-toxic BS reagent No complex procedures	Long reaction time RNA prone to degradation Low-abundance RNA detection challenging	Xue et al. (2020), Zhang et al. (2024), Dai et al. (2024)
UBS-seq	Converts unmethylated cytosine C to uracil U using high-concentration ammonium bisulfite at elevated temperatures	Rapid reaction RNA damage is minor. Low background noise High accuracy	Mapping issues Difficulty distinguishing 5 mC and 5 hmC	Dai et al. (2024)
m5C-RIP-seq	Requires specific antibodies for m5C recognition and binding	Suitable for genome-wide modification detection	Lack single-base resolution and m5C stoichiometry info Fail to identify low-abundance mRNA methylation	Xue et al. (2020), Zhang et al. (2024), Chen et al. (2021b)
miCLIP-seq	Immunoprecipitation based on RNA m5C methyltransferase (specific RCMT) antibodies	Specific analysis of NSUN2-targeted m5C transcriptome structure	Incomplete transcriptome coverage Limited to specific m5C sites	Chen et al. (2021b)
Aza-IP- seq	Covalent complex formation of 5-azacytidine with methytransferase, followed by antibody capture and sequencing	No enzyme engineering required. Suitable for multiple biological systems.	Lacks single-base resolution Potential to alter gene expression May induce toxicity Biased towards short-lived, dynamic RNAs	Bartee et al. (2021), Xue et al. (2020), Zhang et al. (2024)
AWO-seq	TET demethylase converts m5C to hm5C, while original hm5C is not converted to trihydroxythymine	Minimize false positives	Uncertain chemical conversion efficiency Dependent on TET demethylase Inapplicable for transcriptome-wide m5C detection	Selmi et al. (2021)
M5C- tac -seq	Detection at base resolution via TET-assisted oxidation and chemical labeling	Mild reaction conditions Direct m5C detection in low-abundance and low-complexity RNAs	Underestimates true m5C modification levels Suitable for low-input RNA or single-cell samples	Lu et al. (2024)
NSUN enzyme engineering	Mutated NSUN family enzymes form stable covalent bonds with substrate cytosine residues enabling enrichment of modified RNA via immunoprecipitation	Low interference Higher sensitivity	—	Bartee et al. (2021), Hussain et al. (2013)
Machine learning prediction model	Train classifiers based on RNA sequence features to predict m5C sites	Fast and cost-effective	Requires experimental validation and specific selection	Zhang et al. (2018), Jiang et al. (2025c), Dou et al. (2020)
Nanopore Sequencing	Based on changes in current signal	Sequence full-length native RNA molecules Investigate RNA secondary structure Analyze dynamics of RNA metabolism	Dependent on sequence-encoded information Inapplicable for low-abundance, low-content RNA modifications	Wang et al. (2021), Xu et al. (2024), Begik et al. (2021)

### 2.2 Enzymatic system and biological function of m5C modification

Currently, m5C methylation is known to be present on various RNA molecules in multiple cellular organelles, such as mitochondria and ribosomes (Qiu et al., 2023; Bohnsack et al., 2019; Song et al., 2022). For example, m5C modification affects mRNA ribosome biosynthesis and tRNA translation, and is related to the development of a variety of human diseases (Huang L. et al., 2024). The m5C RNA methylation process involves three main enzyme classes, including methyltransferases, demethyltransferases and m5C readers, commonly referred to as “writers,” “erasers,” and “readers,” respectively (Gu et al., 2023; Meng et al., 2024). The effectors differ among RNAs, and this specificity of effectors or modification sites have brought more opportunities and challenges to the pathogenesis and treatment of various diseases (Liu WW. et al., 2024) (Table 2).

**TABLE 2:** M5C modifications in liver disease.

Types	Regulator	Up/Downregulated	Relevant targets	Target RNAs	Disease	Functions	Years	References
Writer	NSUN2	Up	C2017, C131	mRNA pgRNA	Hepatitis B	NSUN2 positively regulates HBV expression and replication	2023	Feng et al. (2023)
Writer	NSUN2	Up	Epsilon element	mRNA pgRNA	Hepatitis B	NSUN2 deficiency reduces the production of HBc	2024	Su et al. (2024)
Writer	NSUN2	Down	EGR1 IFN-β	mRNA pgRNA	Hepatitis B	NSUN2 promotes HBV export The decrease in NSUN2 expression reduces the production of IFN	2024	Ding et al. (2024)
Writer	NSUN2	Up	E2F1	mRNA	Hepatitis C	NSUN2 promotes HCV stability, replication, assembly, and budding; NSUN2 deficiency inhibits HCV RNA replication by upregulating host antiviral immune response genes inhibit HCV RNA replication	2025	Li et al. (2025b)
Writer	NSUN2	Up	Ras pathway	mRNA	HCC	Multiple NSUN2-related genes are involved in oncogenic pathways	2023	Song et al. (2023)
Writer	NSUN2	Up	Wnt signaling pathway SARS2	mRNA	HCC	NSUN2 promotes HCC cell proliferation, migration, and invasion by regulating Wnt signaling	2024	Xing et al. (2024)
Writer	NSUN2	—	CDKN1A	mRNA	MASH	NSUN2 affects the progression of the cell cycle and the process of fat production.	2021	Liu et al. (2021)
Writer	NSUN2	Up	YAP1	lncRNA	CCA	NSUN2 promotes CCA proliferation and metastasis by stabilizing NKILA expression	2022	Zheng et al. (2022)
Writer	NSUN2	Up	G3BP1, MYC	lncRNA	HCC	NSUN2 promotes HCC development by accumulating oncogenic proteins	2020	Sun et al. (2020)
Writer	NSUN2	Up	SREBP2	lncRNA	HCC	NSUN2 drives HCC progression by promoting cholesterol synthesis	2024	Li et al. (2024d)
Writer	NSUN2	Up	PKM2	mRNA	HCC	NSUN2 promotes HCC metabolism and progression by stabilizing PKM2 mRNA	2025	Qi et al. (2025)
Writer	NSUN4	Up	—	—	HCC	NSUN4 promotes proliferation and migration of HCC cells	2022	Cui et al. (2022b)
Eraser	TET2	—	SREBP1	mRNA	AFLD	TET2 regulates SREBP1d protein expression, affecting fatty acid synthesis	2024	Li et al. (2024b)
Eraser	TET2	—	C2017, C131	mRNA	Hepatitis B	TET2 inversely regulates HBV RNA expression	2023	Feng et al. (2023)
Reader	YBX1	Up	MAX		Hepatitis C	YBX1 enhances HCV RNA stability and promotes HCV RNA replication and viral assembly	2024	Li et al. (2024a)
Reader	YBX1	Up	RNF115-DHODH	mRNA	HCC	YBX1 promotes the progression of hepatocellular carcinoma by inhibiting ferroptosis.	2025	Li et al. (2025a)
Reader	ALYREF	Up	—	—	HCC	ALYREF promotes HCC progression by regulating expression of multiple target genes	2023	Xue et al. (2023)
Reader	ALYREF	Up	STAT3	mRNA	HCC	ALYREF promotes HCC cell proliferation, migration, and invasion by activating the STAT3 pathway	2024	Nulali et al. (2024)
Reader	ALYREF	—	YBX2 MO	mRNA	MASH	ALYREF increases the expression of YBX2 protein and inhibits fat formation	2022	Liu et al. (2022a)

Methylation is the formation of a covalent intermediate between a cysteine residue in methyltransferases and a cytosine in the target RNA, which makes the C atom at the C5 position a nucleophilic molecule, binding to the methyl group of S-adenosylmethionine and facilitating the transfer of the methyl group (Bohnsack et al., 2019). Methyltransferases in the m5C RNA methylation are mainly NSUN (NOL1/NOP2/SUN domain) family members (NSUN1-7) and DNMT2 (DNA methyltransferase homolog 2) (Meng et al., 2024). NSUN2 is one of the main m5C mRNA methyltransferases in human cell lines (Yang S. et al., 2023; Zou et al., 2024), which has been the subject of extensive research and found to be linked to the occurrence and development of various diseases. NSUN2 can catalyze m5C modification to enhance the stability of tRNA and mRNA (Li et al., 2023). Lukas et al. confirmed that NSUN3 regulates ESCs cell differentiation by promoting mitochondrial activity, playing a crucial role in determining stem cell fate (Trixl et al., 2018). NSUN4 has a pivotal function in the immune response through the regulation of the stability and transport of mitochondrial double-stranded RNA (dsRNA), thereby maintaining cellular homeostasis (Li D. et al., 2024). NSUN5 plays a significant role in promoting cell growth, enhancing protein translation efficiency, strengthening antioxidant stress resistance and prolonging the lifespan of cells and organisms (Heissenberger et al., 2019). For example, the loss of NSUN5 impairs necortical neuronal layered structure formation and pyramidal cell development (Yuan et al., 2019). Meanwhile, research on the mechanism of NSUN7 in the occurrence and development of liver diseases is relatively scarce, and its function has not yet been fully understood. On the one hand, DNMT2 can act on different m5C sites of tRNA to promote protein synthesis and cell differentiation; on the other hand, it can also act on m5C site of mRNA to participate in the process of cell proliferation and migration (Goll et al., 2006; Schaefer et al., 2010; Tuorto et al., 2012).

Proteins in the ALKBH1 (ALKB homolog 1) and TET (10–11 translocation) family (TET1-3) are known demethyltransferases. The TET family relies on α-ketoglutarate to demethylate m5C to produce 5-hydroxymethylcytosine (hm5C) (Gu et al., 2023), and hm5C can be produced by TET2 and ALKBH1, while 5-formyl cytosine (f5C) can only be formed by ALKBH1, which process is essential for the maintenance of normal mitochondrial function (Kawarada et al., 2017). TET1 ensures the proper completion of DNA repair and cell survival after DNA damage. TET2 has the potential of promoting or suppressing cancer: it can play an inhibitory role in ovarian cancer, prostate cancer, clear cell renal cell carcinoma, while it has a promoting role in low-grade glioma (Gu et al., 2023). Studies have suggested that ALKBH1 mainly targets mRNA, followed by IncRNA, and participates in the development of a variety of cancers via the regulation of various mechanisms.

The biological function of m5C modification depends on the specific recognition of the corresponding reading proteins and subsequent initiation of the regulation of biological processes (Huang L. et al., 2024; Yang et al., 2017; Zou et al., 2020). ALYREF (Aly/REF export factor) and YBX1 (Y-box binding protein 1) are widely studied m5C reading proteins. They promote mRNA splicing by recognizing m5C and regulate correct mRNA output, protein expression and stability, thereby affecting gene expression and post-transcriptional regulation (Cordiner et al., 2023; Pa et al., 2023; Zuo et al., 2023; Wang et al., 2023; Liu et al., 2022a). The role of ALYREF is to recognize and bind to the m5C sites in RNA, thereby facilitating RNA export (Xue et al., 2023). It binds to m5C-methylated mRNA through its cold shock domain to stabilize mRNA, and can also regulate gene transcription and the proliferation rate of related tumor cells (Song et al., 2022; Li et al., 2017; Tao et al., 2023; Chen X. et al., 2019). Recent studies have also suggested that SRSF2 (serine/arginine-rich splicing factor 2) is a splicing factor that recognizes m5C through its unique domain and participates in the splicing regulation of pre-mRNA, thereby ensuring the correct processing of RNA and regulating the diversity of proteins (Ma et al., 2023).

## 3 Physiology and pathology of m5C RNA methylation in the liver

Despite recent advances in the mechanistic understanding of liver development, metabolism and repair processes, liver diseases still represent a significant global morbidity and mortality burden (Trefts et al., 2017). The 2023 update of the Global Burden of Liver disease also pointed out that liver diseases have a high mortality and disability rate, and the main areas of liver disease concern include metabolic dysfunction-associated fatty liver disease (MAFLD), viral hepatitis and liver cancer. Nonetheless, through the knowledge of liver physiology and emerging research targets (Devarbhavi et al., 2023), we can enhance our in-depth understanding of liver physiological and pathological processes. Next, we discuss liver injury and regeneration, liver immune response, lipid metabolism, liver viral invasion, and the terminal outcome of liver lesions from the perspective of the m5C RNA methylation modification.

### 3.1 Hepatic lipid metabolism

**FIGURE 1:**
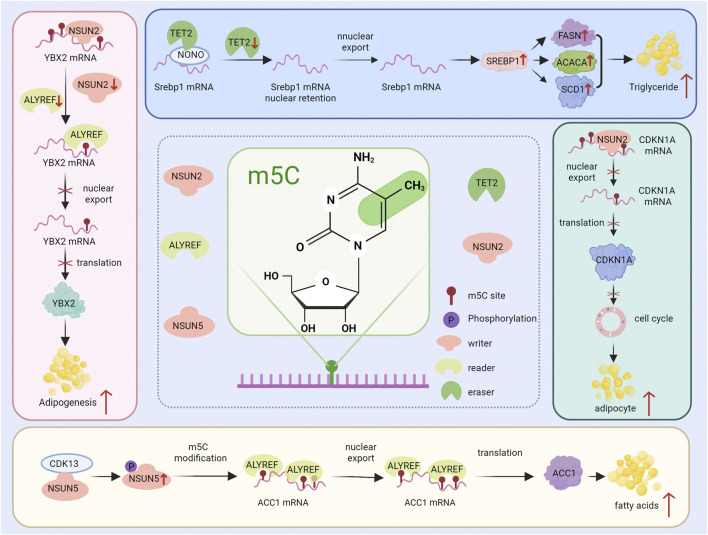
Role of M5C methyltransferase in liver injury. The NSUN2 methyltransferase has a dual mechanism of action: (1) it promotes liver injury by increasing ROS; and (2) it inhibits liver injury by increasing global protein levels. The NSUN5 methyltransferase promotes liver injury through the ferroptosis pathway.

Hepatic steatosis is a liver condition resulting from obesity and metabolic syndrome (Anstee et al., 2019). Liver disease may progress from isolated steatosis to more severe forms such as steatohepatitis, fibrosis and cirrhosis (Saeed et al., 2025; He X. et al., 2025). Currently, it is suggested that the dynamic regulation of m5C is crucial for hepatic lipid metabolism (Li D. et al., 2024) (Figure 1).

ALYREF relies on M5C methylation modification to affect the transport of Y-box binding protein 2 (YBX2) and G protein-coupled receptor Smoothened (SMO) from the nucleus to the cytoplasm and the protein expression, thereby regulating fat production (Liu et al., 2022a). During mitotic clonal expansion (MCE) in early adipogenesis, NSUN2 triggers the m5C modification of CDKN1A mRNA and recruits the reader protein ALYREF to recognize m5C targets, promoting the CDKN1A mRNA shuttle from nucleus to cytoplasm and enhancing its translation. As a result, the cell cycle progression of adipocytes is inhibited (Liu et al., 2021). Cell cycle-dependent kinase 13 (CDK13) promotes the phosphorylation of NSUN5 at Ser327, increases the m5C modification of acetyl-CoA carboxylase (ACC1) mRNA and enhances the stability and nuclear exports of ACC1 mRNA, leading to the upregulation of ACC1 expression and increased lipid deposition (Zhang et al., 2023). This study indicates that regulating the m5C methylation modification may become a new strategy for intervening in diseases related to fat metabolism.

In metabolic-related fatty liver diseases, the regulatory factors m5C have also been observed to have an impact on lipid metabolism. For example, TET2 plays a crucial regulatory role in alcoholic fatty liver disease (AFLD). Specifically, it mediates the demethylation of the 3′UTR of Srebp1 mRNA by binding to the core protein NONO of the heterochromatin. The lack of TET2 disrupts the mechanism of nuclear mRNA retention achieved through the heterochromatin-dependent inverted repeat sequences, promoting the translation of Srebp1 protein, thereby upregulating fatty acid synthesis and regulating liver lipid metabolism and the formation of fatty live. Moreover, TET2 knockout significantly aggravates the disorders of glucose metabolism in mice, such as impaired glucose tolerance and insulin resistance. Excessive glycogen accumulation has been demonstrated to destroy glucose metabolism and promotes the conversion of glycogen to triglyceride, thereby aggravating fatty liver. There is evidence that TET2-mediated Srebp1 mRNA affects epigenetic modifications in lipid metabolism (Li Q. et al., 2024). MAFLD is a pathological condition based on the pathological physiology of fat metabolism. If left untreated, it will lead to a poorer prognosis. Therefore, research on the pathogenesis of MAFLD has gradually increased (Sotoudeheian, 2024). In MAFLD, SREBP1 is also important in regulating the occurrence and development of fatty liver (Han et al., 2015). Therefore, in addition to the mechanism by which TET2 affects MAFLD, we can speculate that TET2 may similarly affect the nuclear and cytoplasmic distribution of Srebp1mRNA in MAFLD and thereby influence liver lipid metabolism. In Metabolic dysfunction-associated steatohepatitis (MASH), the changes in the m5C modification pattern are mainly influenced by lipid metabolism regulation, inflammatory response regulation and cellular stress response, which affect the progression of MASH. For instance, NSUN5 works in conjunction with ALYREF to promote the nuclear export and translation of ACC1 mRNA through m5C modification, accelerating fat accumulation (Zhang et al., 2023). M5C modification can also influence the expression of inflammation-related genes, intensifying liver inflammation and ultimately leading to the promotion of MASH progression to liver fibrosis and cirrhosis (Li D. et al., 2024). NSUN2 regulates the expression of ACSL6 mRNA through m5C methylation modification, thereby modulating the glucose and lipid metabolism disorders in type 2 diabetes. Therefore, by regulating the NSUN2-ACSL6 axis, it may restore the dysregulated glucose and lipid metabolism in the liver (Jiang X. et al., 2025).

### 3.2 Dynamic reprogramming liver damage and regeneration

**FIGURE 2:**
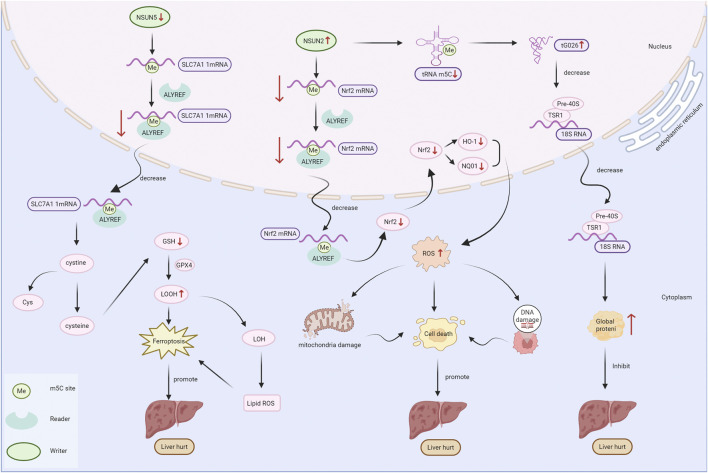
M5C regulatory factors play a role in the formation of fatty liver. The NSUN2/NSUN5 methyltransferases promote or inhibit the expression of related proteins through the ALYREF interpretation protein, thereby promoting fat production. TET2 demethyltransferase facilitates fat production by enhancing the expression of related proteins.

NSUN2 can be localized in the nuclei of liver cells (such as liver parenchymal cells, hepatic sinusoidal endothelial cells and Kupffer cells) under non-stress conditions (Ying et al., 2023). The RNA methyltransferase NSUN2 regulates the Nrf2-mediated antioxidant response through ALYREF-dependent m5C modification, thereby alleviating doxorubicin (Dox)-induced liver cell damage (Huang Y. et al., 2024) (Figure 2). The absence of NSUN2 leads to a reduction in the methylation modifications of m5U and m5C on tRNA, thereby causing tRNA degradation and the production of a large number of protective tsRNAs. These small RNA fragments can significantly enhance the survival rate and proliferation ability of liver cells under oxidative stress and chemical damage, suggesting that NSUN2 and its regulated tsRNA pathway are important regulatory factors for liver injury repair. (Ying et al., 2023) (Figure 2).

Epigenetic modifications play an essential role in liver disease and cellular ferroptosis, which process has received considerable attention in recent years (Huang L. et al., 2024). Ferroptosis caused by the disruption of iron metabolism-related pathways can lead to massive hepatocyte necrosis and a variety of liver diseases (Huang L. et al., 2024; Wang et al., 2019). The m5C RNA methyltransferase NSUN5 methylates the mRNAs of ferritin subunits FTH1 and FTL through the m5C modification, promoting their expression and thereby regulating intracellular iron homeostasis. NSUN5 interacts with mitochondrial heat shock protein TRAP1 to form a complex that jointly regulates the expression of FTH1 and FTL. Knockdown of NSUN5 leads to a reduction in m5C modification of FTH1 and FTL mRNA, decreased protein expression, increased iron accumulation and oxidative stress, and promotes ferroptosis (Liu J. et al., 2022). Another study elucidated the molecular mechanism of hepatocyte ferroptosis during the course of ACLF, and found that it is closely related to m5C RNA methylation and the methylation enzyme NSUN5; in the ACLF model group, the mRNA and protein expression levels of NSUN5 were significantly downregulated. NSUN5 can bind to SLC7A11 mRNA and promote the protein translation of SLC7A11 via m5C methylation modification. When the level of methylation transferase of NSUN5 decreases or its activity reduces, the protein translation of SLC7A11 is inhibited, resulting in a decrease in intracellular glutathione (GSH) levels. This, via the pathway of increased lipid peroxides, leads to cell ferroptosis and promotes the development of ACLF (Huang L. et al., 2024) (Figure 2).

The physiological role of NSUN6 in the liver has also been studied, and the deletion of NSUN6 did not show obvious phenotypic differences in the liver of developing and adult mice; it was found to be unnecessary for organ homeostasis but affecting the reduction-oxidation reaction of the liver in response to external stimuli, especially immune challenges (Wang et al., 2022). NSUN7 and the m5C RNA methylation modification mediated by it help regulate the stability of eRNA, and this mechanism cooperates with the precise regulation of PGC-1α on lysine to finely control the expression of genes related to energy metabolism, enabling a sensitive response to metabolic stress (Aguilo et al., 2016).

The liver has a significant regenerative capacity (Chen Y. et al., 2024). The proliferation of hepatic progenitor cells (HPCs) during chronic liver injury can promote liver regeneration and fibrosis. In the pathological environment after liver injury, the interaction of a variety of cytokines can lead to the activation of hepatic stellate cells (Hsc) and HPCs, resulting in the excessive production of extracellular matrix, as seen in non-alcoholic fatty liver disease, viral hepatitis, etc. (Li et al., 2017; Li B. et al., 2022). YBX-1 can not only negatively regulate the expression of extracellular matrix in HPCs by repressing PDGFR-β transcription but also inhibit the expression of collagen in HPCs by disrupting the PDGFR-β/ERK/p90RSK signaling pathway (Li et al., 2017). YBX-1 can also suppress HPC proliferation and reduce liver fibrosis through tumor protein P53 (Li B. et al., 2022). The M5C demethylase TET2 inhibits the phosphorylation of Stat1, thereby suppressing the activation of macrophages induced by Interferon-γ (IFN-γ), and negatively regulates liver regeneration (Chen Y. et al., 2024).

### 3.3 Liver inflammation

The liver is not only the central metabolic organ but also and the main immune organ of the human body. From an immunological point of view, there are a variety of cells in the liver, such as macrophages (Kupffer cells), lymphocytes (such as natural killer cells, T cells or B cells) and hepatic dendritic cells (DC), which are able to present antigens and produce cytokines and chemokines. They are all key players in initiating and shaping the liver immune response. Certainly, while the activation of immune cells in the liver is crucial for maintaining homeostasis, it can also contribute to liver injury (Heymann and Tacke, 2016).

Macrophages are classified as M1s (proinflammatory), M2 (anti-inflammatory), or Mreg (immunosuppressive). Studies have shown that M1-M2 polarization is strongly correlated with the degree of liver inflammation and repair (Epelman et al., 2014). The infiltration of immune cells such as B cells, CD8^+^T cells, M1 macrophages and M2 macrophages was found in some m5C gene clusters, and a differential expression of NSUN6, TET1 and TET3 between m5C immune subtypes was shown (Yu et al., 2022). NSUN6 and TET2 negatively regulate the recruitment of M2 macrophages and M2-related factors through m5C methylation (Yan et al., 2023; Fan et al., 2024). In addition, in both *in vitro* and *in vivo* experiments, the knockdown of NSUN3 increased the infiltration of M1 macrophages and decreased the infiltration rate of M2 macrophages (Jin et al., 2024).

Ferroptosis-related genes are closely related to immune cells, especially M0 macrophages and regulatory T cells. These genes have an important regulatory mechanism in hepatocellular carcinoma (Zhu et al., 2022). DNA-methyltransferase 3A (DNMT3A) is closely associated with dendritic cells, CD4^+^T cells and B cells, while NSUN6 is closely linked to B cells and CD8^+^T cells, and can regulate the tumor immune microenvironment (Fang et al., 2022). The deletion of NSUN2 in CD4^+^ T cells specifically inhibited Th17 cell differentiation (Yang WL. et al., 2023).

In previous bioinformatics analyses, it was found that m5C is associated with the immune microenvironment of liver cell carcinoma, but the specific mechanism remains unclear (Liu T. et al., 2023; Li D. et al., 2022). The latest research conducted in July 2025 revealed that NSUN2 mediates the 5-methylcytosine (m5C) modification of key glycolytic enzyme (GLUT1, HK2, PFKM) mRNA, enhancing their stability and expression, forming a positive feedback loop, which further improves the glucose uptake ability of tumor cells, aggravating the metabolic restriction and functional impairment of CD8^+^ T cells, thereby promoting tumor immune escape and malignant progression (He J. et al., 2025). NSUN2 enhances the stability and transcriptional activity of the lipid metabolism-related gene SOAT2 by promoting the 5-methylcytosine (m5C) modification of its mRNA. This, in turn, promotes cholesterol synthesis and accumulation. This metabolic reprogramming not only supports the rapid proliferation and invasive ability of tumor cells, but also achieves the evasion of immune surveillance by inhibiting the activity and cytotoxicity of CD8^+^ T cells (Jiang J. et al., 2025).

In the review by Meng et al., the potential roles of m5C methylation regulatory factors in the innate immune pathway of hepatocellular carcinoma (HCC) were summarized. However, these do not represent the direct interaction between m5C methylation regulatory factors and HCC. (Meng et al., 2024). Therefore, the research on m5C methylation modification and liver immunity is still in its infancy. Therefore, researchers need to conduct more in-depth studies on the connections among the liver, m5C methylation modification, and immunity.

### 3.4 HBV, HCV

**FIGURE 3:**
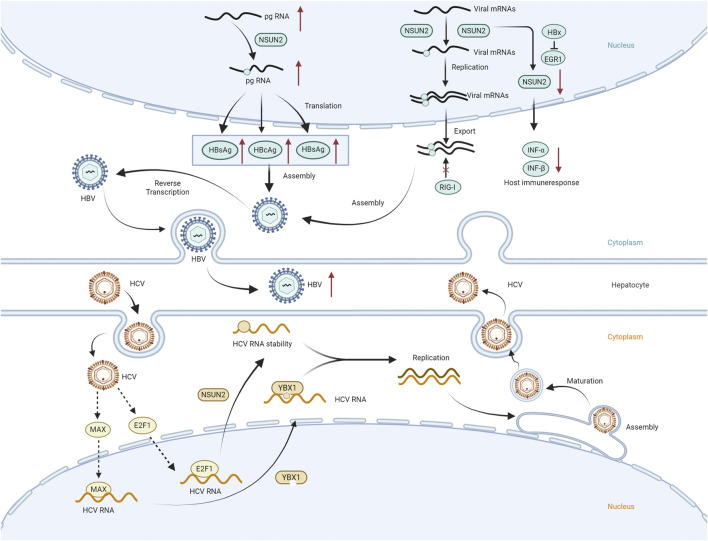
Impact of M5C writers, readers and erasers on HBV/HCV metabolism. The m5C methylation mediated by the NSUN2 methyltransferase plays a positive regulatory role in HBV RNA replication and expression, as well as in modulating HBV RNA replication through immune pathways. The interaction between the YBX1 reading protein and the NSUN2 methyltransferase with HCV RNA m5C methylation promotes viral replication.

The epigenetic modification of hepatitis B virus (HBV)/hepatitis C virus (HCV) has recently become a research hotspot. In the past, many studies addressed the m6A RNA modification in hepatitis virus (Kim and Siddiqui, 2021). Meanwhile, with the development of m5C detection technology, researchers gradually began to explore the role of m5C modification in HBV/HCV replication and better understand the function of m5C methylation in the life cycle of HBV and HCV (Figure 3).

M5C modification mediated by the m5C methyltransferase NSUN2 promotes HBV RNA stability, and knockdown or knockout of NSUN2 results in reduced HBV expression and replication (Su et al., 2024). Feng et al.'s study reached the same conclusion: the absence of NSUN2 leads to a reduction in m5C modification on HBV RNA, thereby negatively regulating HBV expression. On the other hand, m5C demethylase TET2 inversely regulates HBV RNA expression, and the absence of recognition protein YBX1 does not result in significant changes in HBV antigen and RNA levels (Feng et al., 2023). Furthermore, it was found that m5C modifications are primarily concentrated on the epsilon hairpin structure of HBV RNA, and NSUN2 deposits m5Cs on the epsilon RNA element, enhancing the production of viral particles. This is a host-mediated mechanism and may be a target for future antiviral drug development (Su et al., 2024). Another study showed that during HBV infection, the HBx protein inhibits the binding of early growth response 1 (EGR1) to the NSUN2 promoter, leading to a decrease in NSUN2 expression. This reduction in NSUN2 expression decreases the production of interferon-α/β (IFN-α/β), allowing the virus to evade retinoic acid-inducible protein I (RIG-I)-mediated immune responses. However, the decrease in NSUN2 expression enhances viral replication and antigen secretion (Ding et al., 2024).

Zhu et al. first revealed the key regulatory role of the host m5C reading protein YBX1 in the life cycle of HCV in 2024: HCV infection significantly upregulated the expression of the host m5C reader YBX1 through the transcription factor MAX. YBX1 specifically recognizes the m5C modification of the NS5A C7525 site in the NS5A region of the HCV RNA genome by its tryptophan residue 65 (W65), which significantly improves the stability of HCV RNA and promotes HCV RNA replication and viral assembly. The m5C mutation of HCV RNA (such as C7525A) has a negative regulatory effect (Li ZL. et al., 2024). In 2025, building on previous results, the research team used cell and mouse models to find that HCV infection increases the expression of the host m5C writer NSUN2 via the transcription factor E2F1. NSUN2 can regulate HCV RNA by two mechanisms: (1) NSUN2 positively regulates HCV RNA methylation, promoting its stability, replication, and viral assembly and budding; (2) the loss of NSUN2 upregulates the expression of host antiviral immune response genes, thereby inhibiting HCV RNA replication (Li ZL. et al., 2025).

### 3.5 Liver cancer

**FIGURE 4:**
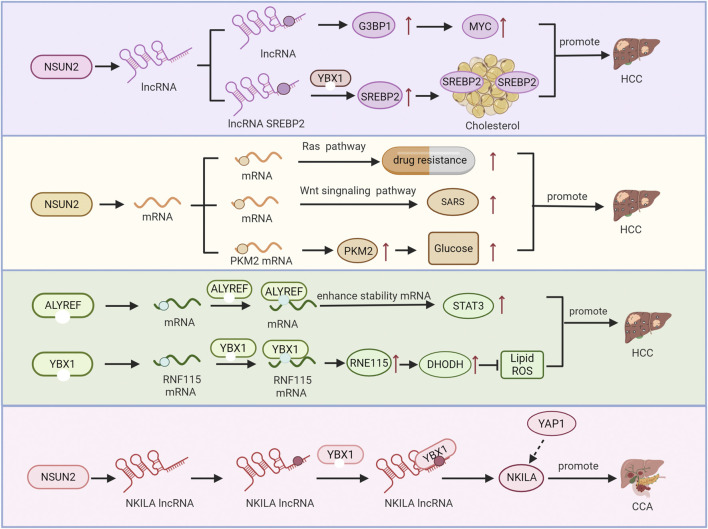
Schematic diagram of regulation between m5C modification and cancer progression. The molecular mechanism mediated by NSUN2, ALYREF and YBX1 for the m5C modification of lncRNAs and mRNAs regulates downstream effectors, thereby promoting cholangiocarcinoma. The progression of hepatocellular Table 1 Approaches for the mapping of m5C in RNA Table 2 M5C modifications in liver disease.

In liver cancer, the expression levels of m5C methylating regulatory factors show significant differences from those in normal liver tissues. These regulatory factors are encoded by genes located on different chromosomal positions. The expression patterns and this unique chromosomal distribution of m5C methylating regulators can affect their expression and function, thereby being closely related to cellular functions (Liu HT. et al., 2023; Zhang et al., 2020). The important role of m5C modification in the development and progression of liver cancer has been identified by epigenetically regulating the function, translation and stability of a variety of RNAs (Pan et al., 2022). For example, NOP2-mediated m5C methylation increases the stability of XPD (Xeroderma pigmentosum gene D) mRNA, thereby inhibiting the proliferation, migration and invasion of HCC cells (Sun and Ding, 2023). In the next part, we will elaborate on the regulatory mechanism of m5C modification in liver cancers such as hepatocellular carcinoma, hepatobiliary carcinoma and hepatoblastoma (Figure 4).

Globally, hepatocellular carcinoma (HCC) ranks as the third most common cause of cancer-related deaths (Shi et al., 2025). In hepaticellular carcinoma, m5C modifications show significant differences in terms of immune cell infiltration and the pathway characteristics, while the occurrence, development and metastasis of disease are closely related to m5C-modified enzyme (Xiao et al., 2023; Liu et al., 2022c). Among them, the m5C modification of NSUN2 plays an important role in HCC, and its expression is closely related to HCC immune regulation and the abundance and distribution of the m5C RNA methylation (Xing et al., 2024). NSUN2 is highly expressed in HCC tissues, and the mRNA m5C modification in these tissues is also higher than that in adjacent normal tissues (Zhang et al., 2020; Song et al., 2023). It has been found that NSUN2 regulates the occurrence and development of HCC through multiple pathways. For example, NSUN2 promotes the proliferation, migration and invasion of HCC cells by regulating Wnt signaling (Xing et al., 2024). Furthermore, it regulates the m5C modification of H19 lncRNA and recruits the G3BP1 oncoprotein to accumulate oncogenic proteins and promote the occurrence and development of HCC (Sun et al., 2020). NSUN2 also promotes HCC progression by regulating the GRB2 mRNA m5C methylation (Song et al., 2023). NSUN2 can further promote the growth and metastasis of HCC by regulating cholesterol metabolism and glycolysis pathway in HCC cells. LINC00618 regulates cholesterol metabolism in liver cancer cells through the ubiquitin-protease-NSUN2-YBX1-SREBP2 axis, ultimately promoting the growth and metastasis of HCC (Li R. et al., 2024). NSUN2-mediated m5C modification at the C773 site in mRNA 3′-UTR can upregulate PKM2 (Pyruvate Kinase M2) to promote glycolysis and HCC progression (Qi et al., 2025). Bioinformatics analysis showed that NSUN4 stimulates the progression of HCC, while the specific mechanism has not been clearly studied (Cui M. et al., 2022).

The m5C reader plays an important role in the occurrence, development, metastasis, and tumor immune microenvironment of HCC. Ferroptosis, previously mentioned in the section on liver immunology, is also involved in HCC development and treatment response (Chen X. et al., 2021; Lei et al., 2022; Gurusinghe et al., 1986). This is mainly related to YBX1, which inhibits ferroptosis via the YBX1-RNF115-DHODH signaling pathway in an m5C-dependent manner and promotes the progression of HCC (Li O. et al., 2025). In addition, the m5C reader ALYREF regulates HCC by directly binding to the 295 HCC cell cycle and apoptosis-related target genes, including the binding of ALYREF to the m5C site in EGFR 3′-UTR to stabilize EGFR mRNA, thereby activating the STAT3 pathway. The upregulation of ALYREF enhanced the proliferation, migration and invasion of liver hepatocellular carcinoma cells (LIHC). Meanwhile, ALYREF deficiency exerted an inhibitory effect on HCC tumors *in vivo*; ALYREF knockdown significantly inhibited the proliferation of HCC cells and increased their apoptosis rate (Xue et al., 2023; Nulali et al., 2024).

Cholangiocarcinoma (CCA) is globally the second most prevalent primary liver cancer, with low survival rates (Bridgewater et al., 2014). Traditional radiotherapy and chemotherapy have no significant effect on the long-term survival rate of CCA patients (Sato et al., 2021). Studies found that NSUN2, by interacting with NKILA (NF-kappa B interacting lncRNA), not only increases the m5C level of NKILA but also promotes the stable expression of NKILA, further facilitating the interaction between NKILA and YBX1. NKILA is related to the TNM staging, lymph node metastasis and distant metastasis of CCA, and can promote the proliferation and metastasis of CCA (Zheng et al., 2022).

Hepatoblastoma (HB), as the most common liver cancer in infants and young children, originates from undifferentiated hepatic progenitor cells (Sharma et al., 2017). YBX-1 can translocate into the nucleus and regulate cell proliferation, adhesion and cancer cell resistance through transcription (Chua et al., 2018; Su et al., 2020). Lau et al. reported that the PDGFR-β inhibitor AG1296 impaired the viability of p53-knockout induced tumorigenic hepatic progenitor cell line (PIL2) in HPCs (Lau et al., 2009). Li et al. showed that HPCs can express both PDGF-β and PDGFR-β. Moreover, YBX-1 can negatively regulate PDGFR-β transcription (Lau et al., 2009); however, the direct role of YBX-1 in HB has not been explored, and more studies are needed to further clarify the regulatory network of m5C modification in HB.

## 4 Therapeutic strategies for liver diseases targeting the m5C modification

It has been clarified that the RNA modification of m5C can be based on complex mechanisms in liver injury, liver inflammation, steatosis, and tumors, which could provide potential intervention points for the treatment of liver diseases, open up new possibilities in drug development, and offer a valuable prediction for the prognosis of liver diseases.

### 4.1 Drug studies targeting the m5C modification

Developing therapeutic approaches for HCC based on m5C modification is highly attractive, showing great promise both in targeted tumor therapy and in improving drug resistance in advanced HCC. For example, through bioinformatics analysis, investigators found that m5C regulatory proteins are closely related to the ErbB/PI3K-Akt axis, and GSK3B (glycogen synthase kinase 3 beta) is an important target of m5C regulators. In the molecular targeted therapy of gastrointestinal (GI) cancers, the compound streptozotocin may be a key candidate for targeting GSK3B (Xiang et al., 2020). NSUN2 and ALYREF-catalyzed methylation of m5C contributes to RNA stabilization and metastasis-associated lung adenocarcinoma transcript 1 (MALAT1) upregulation. In sorafenib-resistant cells, the NSUN2/ALYREF/MALAT1 signaling axis is activated and the upregulation of MALAT1 inhibits sorafenib-induced ferroptosis, thereby driving sorafenib resistance (Shi et al., 2025). In HCC, the GRB2, AATF and RNF115 genes show hypermethylation status, which can participate in carcinogenic pathways. The knockdown of NSUN2 inhibits the cell cycle and significantly reduces the mRNA expression of oncogenes GRB2, RNF115 and AATF. That is, NSUN2 inhibits Ras signaling pathway activation and reduces the levels of Phospho-extracellular regulated protein kinases (p-ErK) in HCC, resulting in an increased sensitivity of HCC cells to sorafenib (Song et al., 2023).

Although no specific inhibitor of m5C RNA regulator has reached the clinical application stage, such treatment has been investigated in hepatitis. MALAT1 is a lncRNA that is aberrantly expressed in sorafenib-resistant HCC cells. The m5C methylation, catalyzed by NSUN2 and ALYREF, enhances RNA stability and leads to the upregulation of MALAT1. The MALAT1 inhibitor MALAT1-IN1 can significantly enhance the efficacy of sorafenib in treating HCC both *in vivo* and *in vitro* (Shi et al., 2025). YBX1 acts as a specific “reader” for m5C modifications at the C7525 site of the viral NS5A gene, enhancing the stability of the viral RNA and promoting the replication of viral RNA as well as the assembly and budding of viral particles. The key amino acid residue W65 is crucial for the function of YBX1. The absence of YBX1 or the application of its inhibitor SU056 can significantly inhibit the RNA replication and expression of viral proteins of HCV. Moreover, the specific mutation of the m5C site in HCV RNA (C7525A) not only reduces the stability and replication efficiency of the viral RNA, but also hinders the co-localization of YBX1 with lipid droplets and viral core proteins, thereby affecting the assembly and release of the virus (Li ZL. et al., 2024). In addition, the treatment of HBV-infected cells with a small nucleotide epigenetic drug, 5-azacytidine (5-AzaC), achieved a significant reduction in HBV replication, but its side effects make it currently unsuitable for clinical application in the treatment of HBV (Christman, 2002). Nonetheless, these studies suggest that derivatives of this nucleotide analogue or other m5C regulator inhibitors could be considered as viable alternatives to current viral reverse transcription inhibitors.

The M5C regulatory factor, when combined with traditional treatments, also showed surprisingly positive effects in recovery from liver damage: during the process of ferroptosis in ACLF hepatocytes, the levels of m5C and m5C methyltransferase NSUN5 were downregulated. NSUN5 may have an inhibitory effect on intracellular ferroptosis in ACLF hepatocytes by directly regulating genes related to the ferroptosis pathway. In addition, Horn Dihuang Jiedu Decoction (NDD) ameliorated ferroptosis in ACLF through the NSUN5-SLC7A11 signaling pathway. These findings collectively suggest that SLC7A11 is a promising target for NSUN5-mediated intervention (Huang L. et al., 2024). Furthermore, research shows that the well-known traditional herb Danshen can protect the liver, reduce liver oxidative stress and improve fatty degeneration and cancer, among other conditions (Ge et al., 2025). Regarding the pharmacokinetic characteristics of traditional medicine, it is possible to perform analysis using the newly developed LC-MS method. As society progresses, the field of drug development is constantly evolving, with traditional drug discovery steps gradually transitioning to network pharmacology (Dai et al., 2022). Especially, the combination of genomics technology and network pharmacology allows us to conduct more comprehensive analyses of drug targets, biological pathways, genes, and related diseases. In addition, the organoid technology holds great potential for understanding the mechanisms of liver diseases and their development, as well as for drug screening and personalized medicine (Liu Y. et al., 2024). Thus, we can combine m5C methylation modification with various traditional technologies such as network pharmacology, traditional Chinese medicine, and organoid technology, and establish a new reference methodology for future drug development.

Furthermore, there have been some recent studies reporting on targeted m5C drugs. Even without considering the liver background, these studies provide valuable guidance for the subsequent development of m5C-targeted drugs for specific liver backgrounds, or can help researchers investigate whether these drugs can also function in the liver. Because some important regulatory mechanisms such as ferroptosis are also common in the liver. For instance, the flavonoid kaempferol is a new m5C-targeting drug. Kaempferol can inhibit the m5C modification level mediated by NSUN7, thereby regulating iron apoptosis in lung epithelial cells. It may play an important role in the treatment of acute lung injury caused by sepsis (Zhang et al., 2025). In addition, by targeting the m5C modification mediated by NSUN2, the NSUN2 inhibitor MY-1B and the FSP1 inhibitor iFSP1 were able to significantly inhibit the survival of acute myeloid leukemia (AML) cells (Ye et al., 2025). Based on the structure of the natural product caerulomycin A, 90 new 2,2′-bipyridine derivatives were synthesized. Compound B19 was identified as the specific target of NSUN3. B19 plays a crucial role in the mitochondrial tRNA methylation of CRC cells by binding to NSUN3, and it regulates mitochondrial function and metabolism (Tang et al., 2024).

### 4.2 Prognostic biomarkers

M5C-related genes can predict the prognosis of hepatocellular carcinoma (Xiao et al., 2023; Liu et al., 2022c). Firstly, the m5C score serves as a biomarker to predict patient responses to immunotherapy and identify potential targeted drugs. For instance, HCC patients with low m5C score are more sensitive to Immune Checkpoint Blockers such as anti-CTLA4 monotherapy. However, pancreatic cancer patients with low m5C score benefited significantly from anti-CTLA4 and anti-PD1 combination therapy (Zhan et al., 2023; Liu P. et al., 2022). In the process of differential expression analysis and Cox regression analysis between normal samples and tumor samples in the TCGA database, it was found that NSUN4 was significantly correlated with poor prognosis for HCC patients (Cui M. et al., 2022). Secondly, the m5C reader has great research significance in the prognosis of HCC. ALYREF expression in HCC mainly affects the level of immune cell infiltration and is not only related to the overall survival time of patients (Shi et al., 2025; Nulali et al., 2024). Pan-cancer analysis showed that ALYREF overexpression was significantly associated with advanced tumor-lymph node metastasis stage and poor HCC prognosis (Xue et al., 2021), so the constructed immune prognostic model could effectively evaluate patients. Therefore, the increased expression of ALYREF may serve as a novel biomarker for HCC diagnosis and prognostic prediction. In experimental studies, ALYREF knockdown significantly inhibited HCC cell proliferation and tumor growth, suggesting that ALYREF may be a potential prognostic marker and therapeutic target (Xue et al., 2023; Nulali et al., 2024). YBX-1 is significantly overexpressed in a variety of cancer types and is associated with poor outcomes, especially in HCC, and YBX-1 can be used as a prognostic indicator for HCC (Li Z. et al., 2024). Risk models are also valuable tools to assess the prognosis of cancer patients (Guo et al., 2022). Using the risk model of m5C regulated genes, it was found that the overexpression of YBX1 gene led to poor prognosis of HCC patients (Li D. et al., 2022); ALYREF and NSUN4 could also be used as carcinogenic indicators of HCC prognosis and were related to immune infiltration in the tumor microenvironment. Similarly, Li’s experiment showed that high expression of YBX1/RNF115 predicted poor overall survival in HCC (Li and Yang, 2022). In 2024, Chen et al. evaluated the association between single nucleotide polymorphisms (SNPS) in m5C modifier genes and overall survival (OS) in patients with HBV-related HCC. It was found that NSUN7 and TRDMT1 may regulate the survival of HBV-related HCC patients after hepatectomy alone or in combination in the Chinese population (Chen B. et al., 2024).

The above studies establish an important link between m5C modification and liver-related diseases, thereby enhancing our understanding of the mechanism of the development and progression of liver-related diseases. This indicates that m5C is not only a promising target for developing therapeutic antiviral and antitumor drugs but also has the potential to evaluate the prognosis of patients through m5C-related genes.

## 5 Conclusion

Due to recent advances in m5C detection technology, m5C RNA methylation are identified at an increasing rate. However, there is still an unmet biological need for new sequencing technologies, and researchers are developing more sensitive and less expensive assays such as UBS-seq, facilitating both research and clinical applications of the m5C RNA methylation. This review highlights the significant impact of m5C modification on liver lipid metabolism, hepatitis virus infection and HCC. However, it is evident that the dynamic development and underlying mechanisms are still poorly understood, especially the regulation of m5C modification in autoimmune liver diseases. Transcriptomic information of each liver disease should be actively collected to fully evaluate the potential mechanisms and dynamic changes of RNA modifications, especially m5C RNA methylation, during the progression of liver diseases. In addition, using mouse models and specific cell lines to explore m5C regulators, inhibitors of m5C regulators can be combined with existing medical methods (Western medicine, Chinese traditional medicine, chemotherapy, radiotherapy), etc., which may lead to more promising results and effective treatments. Additional studies in mouse models are necessary to assess the drugs’ specificity and potential side effects in the treatment of liver diseases.
